# Deadlift-Induced Paraspinal Compartment Syndrome: A Case Report

**DOI:** 10.7759/cureus.62314

**Published:** 2024-06-13

**Authors:** Patrick Kroenung, Philip Zakko, Daniel Park

**Affiliations:** 1 Department of Orthopaedic Surgery, Corewell Health East Beaumont University Hospital, Royal Oak, USA; 2 Department of Orthopaedic Surgery, Michigan Orthopaedic Surgeons, Southfield, USA

**Keywords:** low back pain, fasciotomy, rhabdomyolysis, compartment syndrome, paraspinal compartment syndrome

## Abstract

Paraspinal compartment syndrome is a rare and potentially life-threatening condition. Diagnosis and treatment are often delayed due to a broad differential for back pain, from musculoskeletal to abdominal etiologies. Diagnosis is made with difficulty through clinical picture, laboratory values representative of rhabdomyolysis, advanced imaging, and compartment pressure measurements. Unfortunately, this diagnosis is late; therefore, risks of significant morbidity increase. The mainstay of treatment is emergent fasciotomy of the paraspinal muscles and medical management of rhabdomyolysis. The majority of patients return to baseline functional strength and full range of motion after early treatment. We present a case of severe bilateral paraspinal compartment syndrome that resulted in excisional debridement of necrotic muscle, acute kidney injury, and ileus.

## Introduction

Described as early as 1885, compartment syndrome is a pathoanatomic cascade that leads to muscle and nerve death triggered by local tissue destruction [1]. The inciting tissue insult results in bleeding, edema, and inflammation. All of these increase interstitial tissue pressure within an enclosed space defined by fascial borders. Due to the limited compliance of relatively dense fascia, intramuscular pressure rises rapidly, and low-pressure venous outflow becomes extrinsically occluded. Venous hypertension increases the extravasation of osmotically active solutes, further increasing intra-compartmental pressures. This vicious cycle progressively impedes venous outflow relative to arterial inflow. The resulting reduction in perfusion leads to myoneural ischemia and rapid necrosis of soft tissue within the afflicted compartment. While compartment syndrome is confirmed intraoperatively, the most objective diagnostic test is continuous intra-compartmental compartment pressure monitoring [2]. An intra-compartmental absolute pressure greater than 30 mmHg or delta P less than 30 mmHg (pre-anesthesia diastolic blood pressure - compartment pressure) for greater than two hours confers a 94% sensitivity and 98% specificity for compartment syndrome, with surgical intervention often being performed well before two hours from index measurement [2,3,4]. Commonly affected compartments are the leg, thigh, forearm, hand, gluteal group, and foot. Infrequently, this condition can occur in the paraspinal compartment. Lumbar paraspinal compartment syndrome (LPCS) is an exceedingly rare presentation of compartment syndrome first described by Carr in 1985 [5]. Since then, only 30 additional cases of this condition have been documented [6].

## Case presentation

A 27-year-old African American male presented to the emergency department (ED) with a chief complaint of low back pain. After a period of athletic inactivity, he began a new strength training program in anticipation of starting a semi-professional football season. He developed severe cramping pain in the bilateral flank and lower back, left worse than right, within one hour of deadlifting 325 lbs for an unspecified number of repetitions. At the initial presentation, he denied radicular pain, saddle anesthesia, urinary retention, or urinary/bowel incontinence.

A physical exam showed pain over bilateral paraspinal muscles with reproducible tenderness, no midline tenderness, and a negative straight leg raise. Motor strength and sensation in the extremities were normal. Lumbar radiographs demonstrated all vertebral bodies to be aligned with well-maintained intervertebral disc space and no evidence of fracture. Abdominal CT was negative for renal, ureteral, or bladder calculi without signs of hydronephrosis. The liver, spleen, pancreas, gallbladder, and adrenal glands were grossly unremarkable; however, nonspecific thickening of the left colon suggested mild colitis. Creatine kinase (CK) levels resulted at 111,324 U/L (normal 40-230 U/L), suggesting rhabdomyolysis.

**Figure 1:**
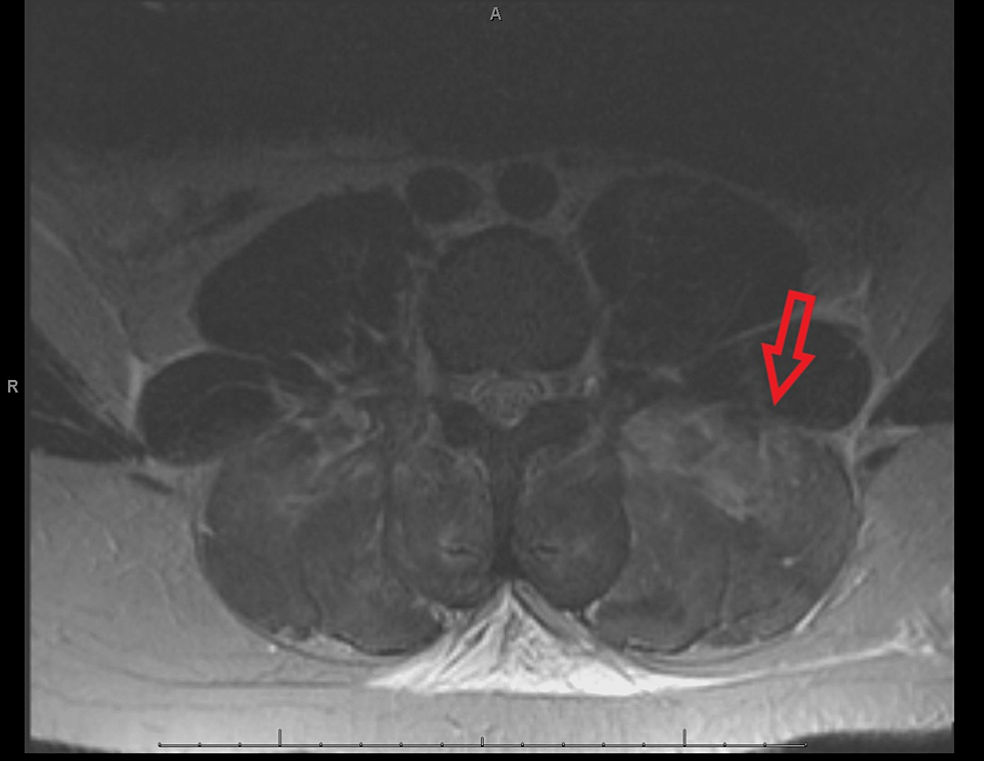
T2-weighted MR sequence demonstrating hyperintense homogenous signal in bilateral paravertebral compartments, left worse than right.

Later on the day of admission, he developed pain-based urinary hesitancy. Repeat labs showed aspartate aminotransferase (ALT) to be 980 U/L (normal 10-37) and CK levels 104,220 U/L. A diagnosis of rhabdomyolysis was established, and aggressive hydration and serial CK labs were initiated. Over the next 48 hours, his lumbar pain became increasingly resistant to IV and oral opioids and muscle relaxers. He was unable to sit up or flex his spine. A Foley catheter was placed for urinary retention, and his abdomen became distended as he could not pass stool or gas. Abdominal X-ray (KUB) demonstrated dilated bowel, with subsequent acute abdomen CT favoring Ileus. He developed radicular symptoms with bilateral leg pain radiating from the lumbar spine down the back of both legs to the ankle, left worse than right. In addition, he had decreased sensation to light touch over the bilateral flank, abdominal wall, and thighs. Due to the development of neurologic deficits, the Orthopedic Spine Surgery Service was consulted 72 hours after the initial presentation to the ED, and appropriate lumbar and thoracic spine MRI sequences were obtained with sedation (Figure 1). Lumbar and thoracic spine MRI demonstrated diffuse and abnormally high T2 signal in the bilateral paraspinal musculature, especially in the lower lumbar spine (Figure 1). There was no evidence of herniated nucleus pulposus, ligamentous disruption, vertebral fracture, or cord compression. Nonspecific subcutaneous edema was present throughout the paravertebral soft tissues (Figure 1).

Based on clinical suspicion and MRI, the patient was taken to the operating room for compartment pressure measurement and likely fasciotomy. Paraspinal compartment pressure measured 90 mmHg on the left and 40 mmHg on the right. The skin was incised with two paraspinal incisions, down to the fascia, from T10 to L5. On the left side, the fascia was incised with immediate muscle herniation, and grossly necrotic paraspinal muscle was debrided accordingly. On the right, the same incisions were performed without profound muscle swelling. The deep musculature was white and pale in appearance without contractility upon stimulation and subsequently debrided. Both wounds were irrigated and covered with a wound vacuum. The patient returned to the operating room five days later for repeat excisional debridement and wound vacuum exchange. Due to persistent ileus and lack of bowel movement for four days, a nasogastric tube was placed by general surgery for bowel decompression.

After the resolution of his AKI and Ileus, four and 11 days post-admission, respectively, his wounds were closed by plastic surgery without necessitating flap coverage. He endorsed well-managed postoperative pain and was cleared for discharge by physical therapy with his spine range of motion within functional limits and full capacity for activities of daily living. At discharge, his CK level was 2,263 U/L, and AST was 125 U/L; both were down-trending. He participated in physical therapy after discharge and, by the end of the year, had returned to weightlifting and full-time manual labor work on an assembly line.

## Discussion

The lumbar paraspinal muscle group lies directly lateral to the vertebral column. The largest muscles within this compartment are the erector spinae muscles. These muscles are arranged as three pillars consisting of the iliocostalis, longissimus, and spinalis from lateral to medial [7]. The entire group is enveloped by a tubular fascial sheath known as the thoracolumbar fascia (TLF). The TLF attaches to the spinous processes posteromedially and transverse processes anterolaterally and forms a contiguous sheet with the transversus abdominis aponeurosis at its lateral margin [3,8]. The mean resting intramuscular pressure within the paravertebral compartment is 3.11 mmHg (range 0-11 mmHg) at rest in the prone position [9]. Paraspinal pressures rise independently with the Valsalva maneuver and they maintain spinal alignment throughout flexion and extension at the hip [5]. The deadlift requires both of these components in order for the lift to be performed in a safe form.

Causes of paraspinal compartment syndrome can also include non-spinal surgery, such as aortic or gastric procedures, direct trauma, vasoconstrictor use, or exercise. Activities such as skiing, rowing, surfboarding, and rugby have been reported as triggering events; however, the most common cause is weightlifting [10,11,12,13]. Thirteen reported cases of paraspinal compartment syndrome in the last decade have involved weightlifting, with the deadlift, as in our case, being the most commonly isolated movement [10,11,12,13].

A progressive increase in lower back pain is the chief complaint in all reported acute paravertebral compartment syndrome cases in conscious patients [4]. Patients develop a washboard rigidity and palpable fullness of the affected paraspinal compartment. Due to the “Bourdon tube effect,” increased intra-compartmental pressure straightens the curvature of the cylindrical paravertebral compartment [12]. This results in the frequently reported loss of lumbar lordosis during acute compartment syndrome. Paravertebral and upper buttock anesthesia are proposed to be pathognomonic for paravertebral compartment syndrome as the sensory branches of the lumbar spinal nerves pass through the paraspinal compartment to provide cutaneous innervation [11]. The presence of lower extremity radicular symptoms, reduced sensation as far as the foot, and absent lower extremity DTRs are infrequently reported but were present in this case [6,12]. Although the ventral rami of the lumbar and sacral spinal nerves travel anteriorly, they are separated from the erector spinae group only intermittently by fascial planes and bound anteriorly by psoas major. Compression and irritation from inflammatory mediators may contribute to radicular symptoms and ventral-sided sensory deficits. Due to the delay in diagnosis, pressures in our patient may have become severe enough to include reduced sensation around the abdomen and thigh from the T8 to L3 dermatomes and bilateral radicular pain. The laterality of severity (left worse than right) corresponded with the respective severity of pressures.

In virtually all cases of LPCS, bowel sounds are absent, presumably from paralytic ileus [10-14]. The proposed mechanism for Ileus in rhabdomyolysis is myoglobin cast aggregation impairing small bowel microcirculation [15]. High doses of opiates and pain with defecation exacerbate the Ileus. Although bowel sounds were not recorded in our case, the clinical presentation, abdominal X-ray, and CT were consistent with ileus.

Laboratory markers that contribute support to a diagnosis of compartment syndrome are those that define rhabdomyolysis. They include elevated CK, myoglobin, and creatinine. A lumbar X-ray will be negative for acute fracture or dislocations but can demonstrate neutral lumbar curvature and bowel dilation. MRI of the thoracic and lumbar spine consistently demonstrates swollen paraspinal musculature with intramuscular homogenous hyperintensity on T2 sequences [10-13,16]. Compartment measurements confirm the diagnosis, and fasciotomy should be performed to prevent further myoneural necrosis, reduce the risk of persistent pain with exertion, and prevent systemic consequences of rhabdomyolysis.

Successful surgical compartment release has been described through percutaneous release, unilateral or bilateral paramedian incisions, and a traditional Wiltse approach [3,13,17,18]. Case-based research demonstrates that regardless of approach, most patients experience resolution of pain and functional limitations within a few weeks of their operation [19]. On the contrary, conservatively managed patients may not resolve lower back pain and could have residual sensory deficits [19].

Due to a recent trend in the popularization of high-volume weightlifting, such as powerlifting and CrossFit, with an increase to over 15,000 registered CrossFit gyms worldwide [20], providers should have heightened awareness of LPCS. Acute paraspinal compartment syndrome must be considered in the differential for patients, especially young men, presenting with back pain out of proportion to the physical exam after posterior chain weightlifting. Diagnosis of this condition is often delayed due to a suspicion of more common causes of acute lower back or flank pain, such as nephrolithiasis, pyelonephritis, herniated lumbar discs, paraspinal muscle strains, fractures, or infectious etiologies of the spine. Paravertebral compartment syndrome has a distinct clinical constellation, which, when combined with a laboratory profile consistent with rhabdomyolysis, warrants emergent advanced imaging with CT or MRI and continuous paravertebral pressure monitoring to make a rapid diagnosis.

## Conclusions

Our patient presented with acute low back pain and labs indicative of rhabdomyolysis following high-intensity weightlifting. He further developed ileus and sensory disturbances of the flank and back, with advanced imaging demonstrating paravertebral swelling. A diagnosis of LPCS was made with intraoperative compartment pressures and subsequent surgical management with fasciotomy resolved his symptoms without permanent functional deficits. Due to limited cases and lack of comparison data, no recommendation can be made on the optimal technique for the surgical release of the paraspinal compartment. If a diagnosis is delayed, further surgical evaluation for the diagnosis and possible treatment of ileus should be initiated. Concurrently, medical management should center on optimizing the treatment of rhabdomyolysis to prevent multi-organ damage. Limitations of this case report are a lack of standardized disability score or functional outcome to quantify recovery from LPCS. In addition, we rely upon chart reviews of physical exams, which are not standardized across the evaluating subspecialties.
